# New perspectives on inoperable early-stage lung cancer management: Clinicians, physicists, and biologists unveil strategies and insights

**DOI:** 10.1016/j.jlb.2024.100153

**Published:** 2024-03-28

**Authors:** Mauro Buono, Gianluca Russo, Valerio Nardone, Carminia Maria Della Corte, Giovanni Natale, Dino Rubini, Lucia Palumbo, Claudia Scimone, Giovanni Ciani, Ida D'Onofrio, Roberta Grassi, Alfonso Fiorelli, Floriana Morgillo, Alfonso Reginelli, Giancarlo Troncone, Salvatore Cappabianca

**Affiliations:** a Department of Precision Medicine, University of Campania “L. Vanvitelli”, Naples, Italy; b Department of Public Health, University Federico II of Naples, Naples, Italy; c Department of Translational Medicine, Università della Campania "L. Vanvitelli", Naples, Italy

**Keywords:** Lung cancer, Oncology, Liquid biopsy, Radiotherapy, Thoracic surgery

## Abstract

This work provides a comprehensive overview of the current landscape of lung cancer, emphasizing the global significance of the disease and the challenges associated with its diagnosis and treatment. The authors highlight the prevalence of lung cancer, with non-small cell lung cancer (NSCLC) and small cell lung cancer (SCC) being the predominant histological subtypes. Advanced-stage diagnosis is common due to the asymptomatic nature of the disease, leading to a systemic treatment approach involving chemotherapy and radiotherapy.The authors discuss the evolution of treatment strategies, with a focus on the emergence of targeted therapies for advanced-stage NSCLC. A panel of predictive biomarkers, both DNA-based (e.g., EGFR, BRAF, KRAS) and RNA-based (e.g., ALK, ROS1, RET, MET), is highlighted as crucial for molecular analysis in diagnostic specimens. While advanced NSCLC patients benefit from targeted therapy, early-stage patients may undergo surgery followed by adjuvant cisplatin-based chemotherapy or stereotactic body radiotherapy (SBRT). The work emphasizes the importance of screening programs for early detection, with a particular focus on the Italian Lung Cancer Screening Network (RISP). RISP aims to recruit high-risk individuals for screening using low-dose computed tomography (LDCT) and implements primary prevention interventions, such as smoking cessation support. The program's objectives include reducing lung cancer mortality, developing a recruitment system for suitable candidates, and integrating radiological, clinical, and molecular data for individual risk profiling. The review also delves into the perspectives of clinicians, physicists, and biologists in the management of lung cancer. Clinicians focus on risk stratification and treatment options, physicists discuss the role of medical physicists in SBRT, and biologists explore precision medicine, biomarkers, and challenges inearly detection.The comparison between surgery and SBRT for early-stage NSCLC patients is discussed, emphasizing the efficacy of SBRT as a non-invasive approach for patients ineligible for surgery. The authors also touch upon ongoing trials addressing the clinical performance of SBRT in comparison to surgery and the challenges posed by preexisting treatment preferences. The physicist's perspective emphasizes the role of medical physicists in lung SBRT, covering aspects from treatment planning to quality assurance. The importance of radiation physics expertise, advanced imaging techniques, image-guided radiation therapy (IGRT), and adaptive radiotherapy is highlighted. Customized models for tumor control and toxicity evaluation, derived from dosimetric analysis, contribute to treatment optimization and patient care. The biologist's viewpoint explores precision medicine in advanced NSCLC treatment, emphasizing the role of somatic alterations as predictive biomarkers. Challenges in early detection are discussed, and the ideal screening tool is proposed to integrate radiological, pathological, and clinical data. Various blood-derived biomarkers and diagnostic assays, such as EarlyCDT-Lung, Nodify XL2, and miRNA-based signatures, are presented as potential tools for early-stage lung cancer detection. In conclusion, the review underscores the multidisciplinary approach required for effective lung cancer management. Advances in early detection, personalized treatment, and the integration of technology and biomarkers offer hope for improving outcomes and reducing the global burden of lung cancer.

## 1 Background and framework

Lung cancer (LC) still represents one of the most leading causes of death worldwide [1]. To date, LC patients are histologically grouped in -non-small cell lung cancer (NSCLC) (accounting for 85% of cases) and small cell lung cancer (SCC) with an incidence of 85.0% and 15.0%, respectively [2]. Among NSCLC patients, adenocarcinoma (ADC) and squamous cell carcinoma (SCC) occurs in 40.0% e 20.0% of cases. Due to asymptomatic evolution, the vast majority of LC patients are diagnosed in advanced stages (III-IV) suggesting systemic treatment approach, including chemotherapy and radiotherapy (RT), by international and national societies [3]. Nowadays, target therapy has been approved in the clinical management of advanced stage NSCLC patients (IIIB– IV) [4]. As regards, a panel of “must test” predictive biomarkers were selected by Societies of College of American Pathologists (CAP), the International Association for the Study of Lung Cancer (IASCL) and the Association for Molecular Pathology (AMP) to assess molecular analysis on diagnostic specimens. This panel includes druggable DNA-based [(*EGFR* (Epidermal Grow Factor Receptoir), *BRAF* (v-Raf murine sarcoma viral oncogene homolog B), *KRAS* (Kirsten Rat Sarcoma Viral Proto-Oncogene)] and *RNA*-based [(*ALK* (anaplastic lymphoma kinase), *ROS1* (protooncogene 1 receptor tyrosine kinase), *RET* (Proto-Oncogene Tyrosine-Protein Kinase Receptor Ret) and *MET* (MET Proto-Oncogene, Receptor Tyrosine Kinase)] biomarkers [5]. Although clinical benefit obtained in advanced setting from the approval of target therapy, stage I and II NSCLC patients may be elected to surgery as best therapeutic option followed by adjuvant cisplatin-based chemotherapy or stereotactic body radiotherapy (SBRT) strategy [6]. At the sight of these critical points, implementations of screening programs are encouraged to identify suspicious lesions in healthy individuals [7] (see Table 1, Table 2, Table 3)

**Table 1:** Clinician's Perspective on Lung Cancer Management. Summary of key points from the clinician's viewpoint, focusing on treatment options, known risk stratification and new perspectives for SBRT in early-stage lung cancer.

Key points	Details
**National Screening Programs**	Improving early diagnosis, increasing demand for SBRT.
**Risk stratification**	- Eastern Cooperative Oncology Group performance status
	-Tumor histology (squamous vs. non-squamous)
	-Nodule size
	-Maximum standardized uptake value (SUVmax)
	-History of lung cancer
	-Age
**New perspectives**	Integrating SBRT with radiomic data and tumor morphology for better outcomes and fewer adverse events.
	Investigating tumor characteristics, genetic profiles, and immunoprofiles for tailored SBRT.
	Upcoming trials exploring adjuvant immunotherapy; personalized follow-up based on risk stratification.

**Table 2:** Key Points from the Physicist's Perspective in Lung Stereotactic Radiation Therapy (SRT). This table provides a concise summary of the physicist's perspective on lung stereotactic radiation therapy (SRT) and the various factors and techniques involved in optimizing treatment outcomes.

Key Points	Details
**Role of Medical Physicists**	Medical physicists play a pivotal role in lung SRT, from treatment planning to quality assurance, ensuring accurate and safe radiation delivery.
**Importance of Radiation Physics Expertise**	Expertise in radiation physics is crucial for creating comprehensive treatment plans tailored to each patient's specific needs. Accurate dose distribution is essential for effective tumor targeting while minimizing exposure to healthy tissues.
**Utilization of Advanced Imaging Techniques**	Advanced imaging techniques such as CT and PET help in precise tumor localization and assessing critical structures.
**Image-Guided Radiation Therapy (IGRT)**	IGRT and advanced delivery technology reduce geometric uncertainties, supporting radiation oncologists in determining treatment fractions and margins.
**Adaptive Radiotherapy**	Adaptive radiotherapy allows treatment modifications to account for anatomical changes during treatment, ensuring treatment accuracy.
**Customized Models for Tumor Control and Toxicity**	Development of local control and toxicity models aids in improving treatment outcomes and minimizing adverse effects. These models consider factors like tumor size, histology, dose distribution, and treatment technique.
**Biomarkers for Predicting Local Control**	Dosimetric analysis, including evaluation of parameters like mean dose, maximum dose, and dose-volume histograms within the tumor and surrounding tissues, helps identify biomarkers correlated with local tumor control.
**Factors Affecting Local Control**	Factors like mean lung dose, V20, V12.4, V13.5, employed fraction scheme, and BED choice influence local control. Higher conformal treatment plans must consider secondary margins and lower prescription doses to minimize risks of distant metastases.
**Importance of Plan Conformity**	Plan conformity indices, such as R100, R50, and D2cm, correlate with overall survival, progression-free survival, and local recurrence-free survival, emphasizing their significance in lung SRT.
**Contribution to Treatment Planning and Decision-Making**	Physicist-derived models aid in treatment planning and decision-making, balancing tumor control and toxicity risk. Continuous development and refinement of models lead to improved treatment customization, reduced toxicities, and better patient outcomes.

**Table 3:** Biologist's Key Points in Lung Cancer Management. This table summarizes the key points from the biologist's perspective on lung cancer management, covering precision medicine, biomarkers, early detection challenges, and various diagnostic assays.

Key Points	Details
**Precision Medicine & Targeted Drugs**	Revolutionize advanced NSCLC treatment; approved target drugs.
**Somatic Alterations as Predictive Biomarkers**	SNVs, INDELs, gene rearrangements in adNSCLC patient stratification.
**Challenges in Early Detection**	Early detection remains a challenge. Ideal tool integrates radiological, pathological, and clinical data.
***EGFR* Drugs in Early-Stage NSCLC**	Clinical efforts on anti-*EGFR* drugs in early-stage NSCLC patients.
**Integration of Radiological and Molecular Data**	Correlation between *EGFR* status and radiological parameters.
**A*LK, KRAS, RET, ROS1 Biomarkers***	Biomarkers linked to specific radiological patterns for different gene aberrations.
**Screening Biomarkers**	Ongoing evaluation of informative, sensitive, specific, easily tested biomarkers.
**Blood-Derived Biomarkers**	Investigate blood-derived biomarkers for early-stage lung cancer detection.
**Diagnostic Assays**	EarlyCDT-Lung assay, mass-spectrometer assay (Nodify XL2), miRNA-based signature.
**cfDNA and Epigenetic Modifications**	Promising role of cfDNA and epigenetic modifications in early-stage lung cancer identification.
**PanSeer Test**	Noninvasive blood test (PanSeer) evaluates cfDNA methylation patterns.

The early detection of lung tumors through screening is a primary objective of the 2021 European Beating Cancer Plan, as it provides the best chances of successful cancer treatment. To implement the lung cancer early diagnosis program for lung cancer patients, in Italy one of the most adopted strategy has been to establish and fund the Italian Lung Cancer Screening Network centers (RISP) program. RISP program aims to compare diagnostic strategies using low-dose computed tomography (LDCT) and primary prevention interventions (such as support for smoking cessation) for high-risk individuals. The RISP program enroll high risk 10,000 individuals to attain a substantial reduction in lung cancer mortality among heavy smokers. This trial evaluates the efficacy of a screening program in participating healthcare facilities and the impact of prevention on life expectancy. The recruitment criteria include healthy individuals aged between 55 and 75 years, who are either heavy smokers or former smokers of no more than 15 years, with an average consumption of 20 cigarettes per day for at least 30 years, and who have not had a tumor for at least 5 years. Specific objectives of RISP include the development of a recruitment system for the most suitable candidates through active collaboration with general practitioners, using a shared database to ensure a continuous flow of information from the evaluation of LDCT results to additional investigations and therapeutic proposals. The individual risk profile will be defined on the basis of epidemiological and radiomic data obtained during the first LDCT (e.g., coronary calcifications, lung damage, respiratory function, chronic inflammatory and immune status). The expected outcomes show decreasing cancer mortality and potentially other smoking-related diseases, achieved by developing an early diagnosis system that uses chest LDCT with variable periodicity tailored on each individual's risk. The results of this randomized clinical trial will validate a personalized screening protocol, with intervals between LDCTs according to individual risk, thereby reducing long-term screening costs. The RISP project also highlights the integration of a pharmacological prevention program to assist high-risk individuals in quitting smoking. Given the increasing age of recently diagnosed LC patients and the anticipated rise in cases in the coming years due to the widespread implementation of the RISP project, it is foreseeable that a significant group of early-stage LC patients may be deemed unsuitable for surgery and might be recommended for non-surgical interventions.

The importance of a multidisciplinary approach in the treatment of lung cancer is crucial in today's era, when we have biological biomarkers that can guide the choice of the most effective therapies. These biomarkers make it possible to personalize treatment for each patient, predict response to specific chemotherapeutic or immunotherapeutic treatments, and steer towards targeted therapies. A multidisciplinary approach involving experts in oncology, radiotherapy, pathology, genetics and other related disciplines, enable to comprehensively assess optimal treatment planning for lung cancer patients. improving the patient's chances of therapeutic success and quality of life.

The synergy among the different specialists allows for a comprehensive and customized clinical administration of tumor patient. Each team member brings his or her own unique expertise and perspective, promoting a comprehensive assessment of clinical cases. Multidisciplinary tumor board guarantee that every aspect of treatment is considered, providing the patient with the most effective and safe therapeutic option. In a context where the complexity of medical conditions is constantly increasing, the presence of a multidisciplinary team becomes crucial in order to meet the challenges and improve clinical outcomes. In our work we explain same innovative aspect of lung cancer treatment.

In early-stage LC treatment, surgery remains the primary option, in particular when the tumor is localized and can be completely removed [[8], [9], [10]]. Surgery offers the opportunity to directly remove the tumor from the lung [10]. However, it is important to note that in some cases, especially when surgery is not feasible due to the tumor's location or size, radiation therapy emerges as an equally effective alternative and, in specific subsets, may be associated with fewer side effects compared to surgery [[11], [12], [13]]. Radiation therapy can be adopted before surgery to reduce the tumor size (neoadjuvant) or as the primary treatment when surgery is not appropriate [12,13]. The comparable effectiveness of radiation therapy underscores the need for a personalized approach in determining the best therapeutic strategy, considering the specific characteristics of the tumor and the patient's conditions. The choice between surgery and radiation therapy will be based on a comprehensive assessment by the medical team, aiming to maximize therapeutic benefits, improve the patient's quality of life, and minimize side effects [13].

The use of radiation therapy becomes particularly relevant when surgery is deemed impractical due to various circumstances, such as in cases of inoperable patient. In these situations, radiation therapy stands out as a preferred therapeutic option, as it allows tumor treatment without the need for invasive surgical procedures. Radiation therapy can be tailored to suit the specific conditions of the patient and may be employed to shrink the tumor, alleviate symptoms, or to control tumor growth. This approach provides a safer and more suitable solution for patients who, for various reasons, are not suitable candidates for surgery. The decision to opt for radiation therapy in cases of inoperability is always guided by the aim of maximizing therapeutic benefits while minimizing the risks associated with treatment, all while keeping the well-being and quality of life of the patient at the forefront of medical considerations [12,14].

Conducting randomized controlled trials comparing surgery to non-surgical treatments for cancer has been notoriously challenging due to difficulties in achieving equipoise and addressing preferences among both patients and providers. The debate over the comparative efficacy of Stereotactic Ablative Radiotherapy (SABR) versus surgery for early-stage LC is no exception to this challenge. Attempts to conduct prospective randomized controlled trials aimed at determining a definitive preference between surgery and SABR have faced difficulties in recruitment, and the analysis combining data from prematurely closed original STARS and ROSEL studies has sparked considerable debate among thoracic oncologists [[15], [16], [17]]. Despite efforts in other comparative effectiveness studies to mitigate confounding factors, inherent limitations persist, leading to polarized opinions [[18], [19], [20]].

Stereotactic Body Radiation Therapy (SBRT) is considered the best treatment strategy in NSCLC patients that could not access to surgical approach. However, prospective randomized trials have failed demonstrating clinical performance in NSCLC patients after surgery and stereotactic ablative radiotherapy [[15], [16], [17]]. Ongoing trials (VALOR-NCT02984761 and STABLEMATES-NCT02468024) aim to address this gap, but it has been documented that preexisting treatment preferences pose a challenge to recruitment and randomization in enrolled patients.

Despite the benefits of both surgery and SBRT, patients undergoing either treatment are affected by high-risk percentage of locoregional and distant recurrence from primary site. Early-stage NSCLC patients with distant metastasis (DM) treated with SBRT, are affected by greatest risk, ranging from 19% to 39% (RTOG 0618, RTOG 0813) of locoregional disease [21,22].

In operable NSCLC patients with tumor sizes ranging from T1 to T2, no lymph node involvement (N0), or metastasis (M0), and tumor diameter under SBRT administration (54 Gy in 3 fractions) not exceeding 5 cm, 4-year primary tumor control and local control rates of 96% (95% CI, 83%–100%) was observed. (RTOG 0618) [21] In addition, PFS of 52.2% (RTOG 0618) [22] in NSCLC patients treated with SBRT was evaluated.

In this rapidly evolving scenario, national level screening programs improves early diagnosis rate in lung cancer patients. This strategy, combined with the progressive aging of the population, may lead to a higher demand for stereotactic treatments. Therefore, it has become crucial to identify higher risk of developing distant metastasis patients suggesting a personalized approach. Unlikely, the calculation risk of recurrence disease can be stratified on the basis of histologic data (e.g., lymphovascular invasion and occult N2). NSCLC patients treated with SBRT are grouped into high or low risk groups taking into account clinical variables like as age, Eastern Cooperative Oncology Group performance status, tumor histology (squamous versus non-squamous), nodule size, maximum standardized uptake value (SUVmax), and history of lung cancer, or nomograms that sum several variables [23,24] As regards, the implementation of SBRT technique *in house* integrated with radiomic data and morphological pattern of tumor cells allows the best clinical administration of tumor patients in terms of clinical outcome and adverse events (AEs) [23].

Furthermore, upcoming results from trials investigating adjuvant immunotherapy following SBRT (PACIFIC4-NCT03833154; ISABR-NCT03110978) [25,26], are expected to shed light on the potential benefits in the elderly individuals with multiple comorbidities. In this context, adjuvant chemotherapy is widely discouraged as therapeutical option. However, the availability of less toxic and more effective systemic treatments, like as immunotherapy and targeted therapies, which have also been tested in the adjuvant setting, represent a clinical valuable therapeutical option in high-risk metastatic NSCLC patients. Moreover, for the NSCLC patients not eligible to adjuvant, personalized follow-up trails based on risk stratification can be assessed. Early identification of any metastases selects patients not treated with systemic therapies to benefit from local ablative treatments, whether for oligometastatic or pluri-metastatic progressions.

Recently, the identification of prognostic and predictive factors has led to a reassessment of treatment choices. Tumor characteristics, genetic and immune-profiles of patients are currently investigated to identify individuals who may obtain the greatest benefit from SBRT.

In a state-of-the-art hospital setting, where the use of radiation for cancer treatments is crucial, the figure of the medical physicist assumes a central role. The medical physicist is responsible for the planning, implementation and precision control of all radiotherapy treatments. His presence ensures that radiation is administered safely and accurately, with the most appropriate technique depending on the location and size of the disease, while minimising damage to surrounding healthy tissue. In this way, thanks to his expertise, the medical physicist also helps to ensure that radiotherapy treatments are effective, maximizsing the probability of success in treating the disease and reducing possible toxicities.

The remarkable clinical outcomes of SBRT [27] have been made possible through a multidisciplinary approach involving highly skilled personnels and advancements in upgraded radiological technologies. To date, an unmet clinical need consists in the lack of standardized procedures to clinically administrate lung patients. Medical physicists play a pivotal role in the entire process of lung SBRT, from treatment planning to quality assurance, ensuring the accurate and safe delivery of radiation to the target area. Expertise in radiation physics is also crucial for a comprehensive treatment plan tailored to each patient's specific needs, with the accurate calculation of dose distribution ensuring effective tumor targeting with a highly conformal dose distribution while minimizing radiation exposure to healthy tissues through a dramatic dose falloff [28]. Advanced imaging techniques such as computed tomography (CT) and positron emission tomography (PET) allows to precisely localize tumor lesion and surrounding anatomical structures, while image-guided radiation therapy (IGRT) and advanced delivery technology reduce geometric uncertainties supporting radiation oncologists for the total number of treatment fractions and margins. Conventional procedures like as deep breath inspiration, gating, or tracking, combined with image guidance, enable the reduction of margins from the clinical target volume (CTV) to the planning target volume (PTV) [29].

Lung tumors can exhibit significant anatomical modifications derived from biological and clinical factors including tumor regression, weight loss, or respiratory motion. Adaptive radiotherapy allows treatment modification in accordance with variations observed during treatment. In this scenario, medical physicists and integrated treatment team is required to assess these modifications, implement plan adaptations, and ensure continued treatment accuracy throughout the course of therapy [30].

Furthermore, medical physicists provide a significant contribution in tumor control and toxicity evaluation by using customized models. Recently, the development of local control and toxicity models has significantly contributed to improve treatment outcomes minimizing potential adverse effects. On one hand, local control models, as statistical tools depending on various factors such as tumor size, histology, dose distribution, and treatment technique, can provide valuable insights into the likelihood of achieving local tumor control. Clinical factors like as tumor volume, site, and biological parameters are integrated enabling the selection of appropriate treatment strategies and optimize dose prescriptions and fractionation scheme for each patient. This approach improves the customization for planning treatment and ensure the delivery of an adequate radiation dose to eradicate the tumor minimizing recurrence risk [[31], [32], [33], [34], [35]].

On the other hand, toxicity models allow the prediction of the probability and severity of treatment-related side effects (radiation pneumonitis, chest wall toxicity, etc.) likewise considering factors such as the dose distribution to critical structures, patient-specific characteristics, and treatment technique. These tools can help the radiation oncology team to identify patients who may be at higher risk of experiencing adverse effects and develop personalized treatment plans to minimize toxicities [[36], [37], [38], [39], [40], [41], [42], [43], [44], [45], [46], [47], [48], [49]].

The assessment and prediction of local control outcomes in lung tumor patients undergoing stereotactic radiotherapy can be made by biomarkers provided by dosimetric analysis: the meticulous evaluation of dosimetric parameters, such as mean dose, maximum dose, dose-volume histograms, and homogeneity indices within the tumor and surrounding healthy tissues can let clinicians to identify specific biomarkers that correlate with local tumor control. According to the AAPM reporting and several other studies [[36], [37], [38], [39], [40], [41]], mean lung dose is considered a significant predictor of symptomatic radiation pneumonitis. Also, V20, V12.4 and V13.5 stand as endpoint for symptomatic radiation pneumonitis [42,43].Local control in biophysical models is associated to several factors, such as the employed fraction scheme and BED choice [31,32]: the available literature indicates a potential dose–response relationship up to doses consistent with at least a BED10 of 100 Gy at isocenter and potentially to a higher level [31,[33], [34], [35]]. Furthermore, tumor size and position can affect the risk-adapted dose-fractionation decision process [50].

Higher conformal treatment plans, with steeper dose gradients, can worsen tumor control, as showed by Diamant et al., who suggested secondary margins in the region of 2 cm around PTV, with a lower prescription dose to consider microscopic disease extension and to reduce the risk of distant metastases [51].

Plan conformity plays a crucial role in local control; indices such as R100 (volume receiving the 100% dose/volume (PTV)), R50 (volume receiving the 50% dose/volume (PTV)) and D2cm (maximum dose to any point 2 cm from the edge of the PTV) are supposed to be correlated to overall survival, progression free survival and local recurrence free survival [52].

These two models provide valuable information during the treatment planning and decision-making process in lung SRT, enabling the identification of optimal treatment strategies that balance tumor control and the risk of toxicity, to achieve the desired treatment outcomes while minimizing potential side effects. The continuous development and refinement of these models contribute to advancements also in the lung stereotactic radiation therapy, resulting in valuable insights into treatment efficacy and potential toxicities, which can be translated into optimized treatment customization, minimized toxicities, and improved patient care ultimately. Additionally, dosimetric analysis-derived biomarkers aid in identifying patients at higher risk of local recurrence, allowing for early intervention and tailored treatment strategies, as well as enhancing the precision and success of lung tumor radiation therapy and leading to improved local control and better patient outcomes.

**Fig. 1:**
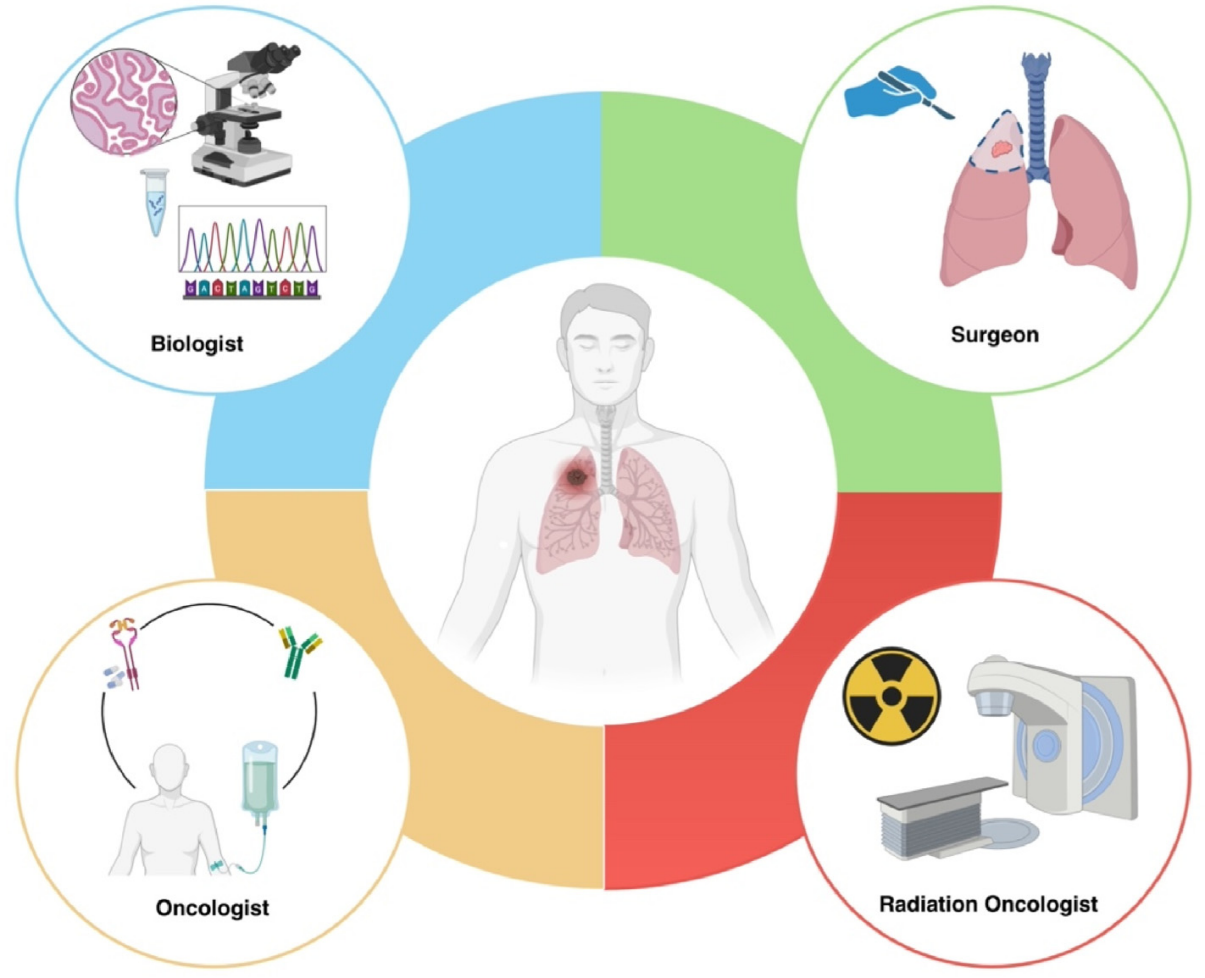
Collaborative Efforts in Lung Cancer Research: Radiation Oncologists (Physician and Medical Physicist), Biologists, and Surgeons unite to unravel the complexities of disease progression and treatment strategies.

With the advent of target therapies, understanding genetic mutations molecular alterations in key actors for cell replication has become crucial. Identifying these specific genetic alterations makes it possible to identify patients who can benefit from targeted and personalized treatments. The use of target can greatly increase the chances of a successful treatment outcome, while reducing undesirable side effects. In this context, the role of the biologist and the molecular pathologist becomes crucial. These healthcare figures are essential to morphologically identify tumor patterns and evaluate recurrent predictive molecular alterations in diagnostic routine samples from lung cancer patients. Their collaboration with the clinical team contributes to ensuring personalized and effective treatment for patients with oncological diseases (Fig. 1).

Nowadays, precision medicine has revolutionized therapeutical scenarios for advanced stages NSCLC patients [4,53].As regards, a novel generation of target drugs have been approved by international societies in the clinical management of adNSCLC patients. Particularly, randomized clinical trials established the predictive role of somatic alterations, including SNVs, INDELs, gene rearrangements in predictive biomarkers that play a key role in clinical stratification of adNSCLC patients [4,53]. Although these encouraging advantages in the clinical management of adNSCLC patients screening programs, able to detect early-stage lesions, early detection may represent an opening challenge for the management of lung cancer patients [54].As regards, an ideal screening tool should integrate radiological, pathological and clinical data in order to provide diagnostic, prognostic and predictive assumptions for each NSCLC patients [7]. Firstly, clinical efforts were carried out to evaluate the role of anti *EGFR* drugs in early stage (II-IIIA) NSCLC patients [55]. In the same way, Lee et al. investigated integration between radiological and molecular data in stage I NSCLC patients after curative resection [56]. In details, a statistically significant *EGFR* overexpression was found in patients with SUVmax >5.0 (p < 0.0001), diameter >2.43 cm (p < 0.0001), and with ground glass opacity ≤50% (p = 0.0073). Moreover, multivariate analysis was also able to distinguish *EGFR* exon 21 p.L858R mutations and exon 19 deletions on the basis of radiological parameters (ground glass was prominent in exon 21 positive NSCLC patients) [57]. In a prospective series of NSCLC patients, Rizzo et al. revealed a positive trend in *EGFR* mutation rate associated with radiological derived signature including pleural retraction, small lesion size and absence of fibrosis. Noteworthy, *ALK* rearrangements were remarkably detected in pleural effusion NSCLC patients while *KRAS* hotspot mutations were observed in NSCLC patients showing specific radiological pattern (round-shape, nodules in non-tumor lobes) [58]. In a similar experience, *RET* and *ROS1* aberrant transcripts were linked to specific radiological models. Briefly, *ROS1* druggable rearrangements were frequently identified in peripheral lesions while *RET* gene fusions were observed in spiculated borders with rare cavitation or calcification [59]. In this scenario, the identification of clinically relevant screening biomarkers is currently under evaluation. A clinical routinely approved biomarker should be informative, sensitive, specific, easily purified and tested. In this scenario, liquid biopsy has demonstrated clinically valuable results in the clinical management of early-stage lung cancer patients [[60], (a), (b)]. Within the term liquid biopsy, a plethora of biological fluids (blood, saliva, urine, CFS, tears) are included [61]. To date, liquid biopsy is yet approved by international societies in the clinical stratification of advanced NSCLC patients eligible to target therapy. Among several analytes in torrent blood, ctDNA, a small fraction of cfDNA, is the only diagnostically available analyte for molecular analysis in clinical practice [62]. In addition, a wide series of blood derived biomarkers are currently investigated thanks to reproducible, saving money isolation strategies [7]. Moreover, other biological fluids derived biomarkers including saliva and urine may play a potential role in early-stage lung cancer detection. In this scenario, commercially available diagnostic assay has been developed to molecularly classify indeterminate lung lesions [7]. Firstly, EarlyCDT-Lung (OncImmune) assay is based on seven mAB panel able to bind p53, CAGE, NY-ESO-1 (CTAG1B), SOX2, GBU4–5, HuD, and MAGE-A4 proteins from torrent blood. This test highlighted a technical sensitivity and specificity of 90.0% and 40.0%, respectively [63]. In a randomized clinical trial enrolling 12.000 high risk individuals, Early CDT-Lung test showed that 58.9% lung cancers from experimental cohort were detected in advanced stage (III/IV) compared with 73.2% from the control arm [63]. A second approach was carried out adopting a mass-spectrometer assay for the analysis of a blood-derived protein panel able to distinguish between benign and malignant lesions (Nodify XL2, Biodesix). A series of n = 178 individuals (≤50% cancer risk index) were investigated in observational clinical trial (PANOPTIC). Among them, lung cancer was diagnosed in 16.0% of patients. Interestingly, clinical and molecular data integration highlighted 97.0% (CI, 82–100) and 44.0% (CI, 36–52) of sensitivity and specificity, respectively, grouping malignant and benign lesions [64]. In addition, a miRNA-based signature comprising n = 24 differentially expressed circulating miRNA (MSC) highlighted valuable technical accuracy in preliminary validation trials [65]. In this scenario, circulating-free DNA (cfDNA) also demonstrated a promising role in the identification of early-stage lung cancer patients. Particularly, an observational trial investigating how modification in cfDNA level from torrent blood may drastically impact on the cancer risk. This approach was sustained by an ultra-deep NGS system (CAPP-Seq) able to detect low abundant cfDNA in blood samples. Moreover, bioinformatic tool (Lung-CLiP) was integrated in NGS analysis filtering cancer-related somatic mutations from clonal hematopoiesis derived molecular alterations [66]. Remarkably, epigenetic modifications also play a pivotal role in driving cancer development. A noninvasive blood test (PanSeer) evaluates cfDNA methylation pattern in a series of asymptomatic individuals (n = 605), cancer patients (n = 223) including primary tumor and normal matched tissues. Of note, PanSeer enables to identify cancer in 95% (95% CI: 89–98%) of asymptomatic individuals [67].

## 2 Conclusions

In conclusion, this comprehensive review provides a multifaceted perspective on inoperable early-stage lung cancer, highlighting the critical importance of early detection and personalized treatment strategies. Lung cancer remains a formidable global health challenge, with NSCLC and SCC as the primary culprits, often diagnosed in advanced stages. The advent of targeted therapy has reshaped treatment options, offering hope to advanced-stage NSCLC patients with specific biomarkers. At the same time, the introduction of screening programs, such as the Italian Lung Cancer Screening Network (RISP), is a pivotal step forward in early identifying lung cancer more manageable stages. RISP's integration of radiological, clinical, and molecular data exemplifies the comprehensive approach needed for optimal patient management. The comparison between surgery and SBRT for early-stage NSCLC patients underscores the importance of treatment selection, especially in those with comorbidities. While both options show promise, ongoing prospective trials will provide further insights into their relative effectiveness. The involvement of medical physicists in lung SBRT is indispensable for precision and safety. Cutting-edge imaging techniques and adaptive radiotherapy contribute to more accurate and tailored treatment plans. Dosimetric analysis-derived biomarkers aid in predicting local control and minimizing adverse events, enhancing the quality of care. From a biological perspective, precision medicine has transformed the landscape of NSCLC treatment. Somatic alterations serve as predictive biomarkers, guiding clinical decisions. Early detection remains a challenge, but ongoing research into blood-derived and other biomarkers offers promise in improving diagnostic accuracy.

In sum, this review underscores the importance of collaboration across disciplines in the fight against lung cancer. With advances in early detection, personalized treatment, and the integration of cutting-edge technology and biomarkers, there is hope for improving outcomes and ultimately reducing the burden of this deadly disease.

## Declaration of competing interest

The authors declare the following financial interests/personal relationships which may be considered as potential competing interests:

Mauro Buono reports was provided by University of Campania Luigi Vanvitelli. If there are other authors, they declare that they have no known competing financial interests or personal relationships that could have appeared to influence the work reported in this paper.
